# Partnership for Healthier Asians: Disseminating Evidence-Based Practices in Asian-American Communities Using a Market-Oriented and Multilevel Approach

**DOI:** 10.2196/resprot.5625

**Published:** 2016-06-16

**Authors:** Karen Kim, Michael Quinn, Edwin Chandrasekar, Reena Patel, Helen Lam

**Affiliations:** ^1^ The University of Chicago Comprehensive Cancer Center Chicago, IL United States; ^2^ The University of Chicago Department of Internal Medicine Chicago, IL United States; ^3^ Asian Health Coalition Chicago, IL United States

**Keywords:** Evidence-based practice, community-based participatory research, Asian Americans, dissemination and implementation, community health advisors

## Abstract

**Background:**

One of the greatest challenges facing health promotion and disease prevention is translating research findings into evidence-based practices (EBP). There is currently a limited research base to inform the design of dissemination action plans, especially within medically underserved communities.

**Objective:**

The objective of this paper is to describe an innovative study protocol to disseminate colorectal cancer (CRC) screening guidelines in seven Asian subgroups.

**Methods:**

This study integrated a market-oriented Push-Pull-Infrastructure Model, Diffusion of Innovation Theory, and community-based participatory research approach to create a community-centered dissemination framework. Consumer research, through focus groups and community-wide surveys, was centered on the adopters to ensure a multilevel intervention was well designed and effective.

**Results:**

Collaboration took place between an academic institution and eight community-based organizations. These groups worked together to conduct thorough consumer research. A sample of 72 Asian Americans participated in 8 focus groups, and differences were noted across ethnic groups. Furthermore, 464 community members participated in an Individual Client Survey. Most participants agreed that early detection of cancer was important (434/464, 93.5%), cancer could happen to anyone (403/464, 86.9%), CRC could be prevented (344/464, 74.1%), and everyone should screen for CRC (389/464, 83.8%). However, 35.8% (166/464) of participants also felt that people were better off not knowing it they had cancer, and 45.5% (211/464) would screen only when they had symptoms. Most participants indicated that they would screen upon their doctor’s recommendation, but half reported that they only saw a doctor when they were sick. Data collection currently is underway for a multilevel intervention (community health advisor and social marketing campaign) and will conclude March 2016. We expect that analysis and results will be available by June 2016.

**Conclusions:**

This study outlines a complementary role for researchers and community organizations in disseminating EBP, and incorporates social interactions and influences to move individuals from simple awareness to decisions towards positive action.

## Introduction

One of the greatest challenges facing health promotion and disease prevention is translating research findings into evidence-based practices (EBP). Despite significant accomplishments in basic, clinical, and population health research, a wide gap persists between what we know and what we do. Failing to translate knowledge into practice is costly and harmful; it leads to overuse of ineffectual care, underuse of effective care, and errors in execution [1]. In recent years, there has been a growing effort to bridge the knowledge and practice gap, yet there is a limited research base to inform the design of dissemination action plans, resulting in slow and uneven adoption of EBP [2-6]. EBP is essential in health care, since it provides direction and rationale for guiding health behaviors, decision-making, and treatments [7]. Based on marketing and diffusion theories, many researchers agree that a fundamental obstacle to successfully disseminating EBP to a wider audience is the lack of systems and infrastructure to carry out marketing and distribution [8-12]. Marketing and distribution systems bring products and services from development to use through a system of intermediaries [13]. These intermediaries identify potential users, promote the product to them, provide them with easy access to the product through multiple channels, and support the product after purchase [14,15]. Building a successful marketing and distribution system to bring EBP to medically underserved communities has great potential to reduce unnecessary disease burden.

Asian Americans (AAs) are the fastest growing minority group in the United States, and there are approximately 17.3 million AAs nationwide [16]. More than 65% of AAs are foreign-born with greater than 30% having limited English proficiency [17]. AAs are the first and only racial/ethnic group to experience cancer as the leading cause of death [18]. Although colorectal cancer (CRC) is the third most common cancer in the United States, it is the second most common cancer among AAs [19,20]. Disturbingly, 50% of new cases diagnosed yearly in the United States could have been avoided with routine CRC screening [21,22]. Although there was a significant increase in CRC screening in the overall US population between 2008 and 2010, AAs remained the only racial/ethnic subgroup without any improvement [23]. In addition, important differences exist in knowledge, attitudes, and beliefs among Asian subgroups [24]. In this project, we aim to develop a community-centered dissemination system for EBP in Asian communities.

## Methods

### Conceptual Framework

**Figure 1:**
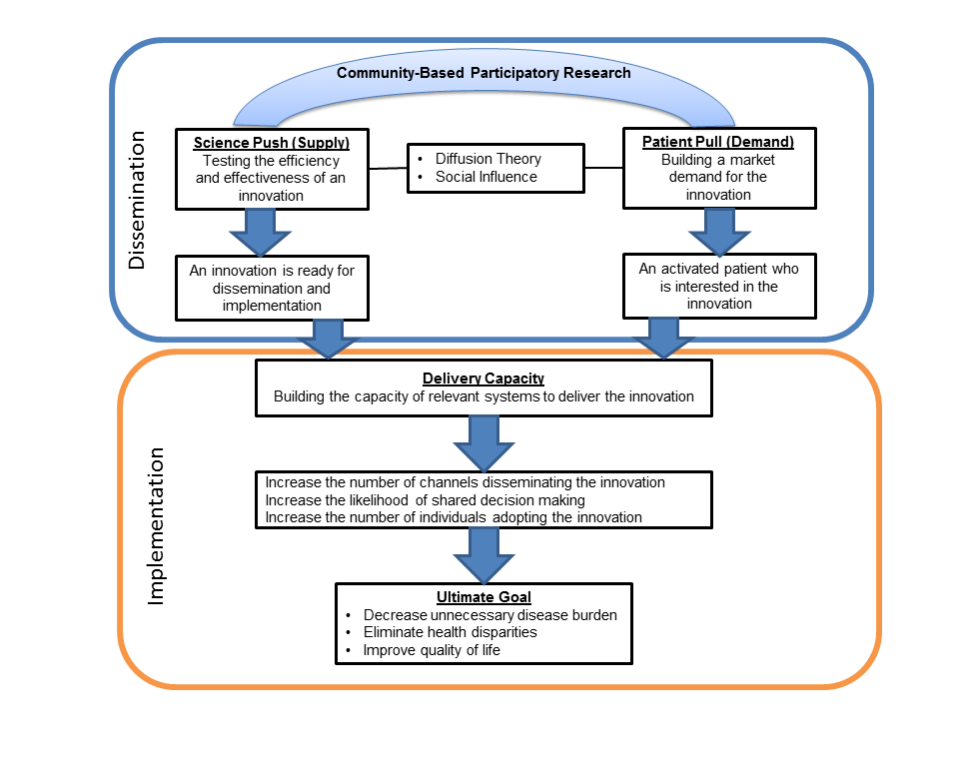
A community-centered dissemination framework for evidence-based practice.

We integrated a market-oriented Push-Pull-Infrastructure Model [25] with Diffusion of Innovations Theory [26], and Community-Based Participatory Research (CBPR) approach to create a community-centered EBP dissemination framework (Figure 1). The Push-Pull-Infrastructure Model implies that a sole emphasis on pushing (supplying) knowledge from science is ineffective. The supply of knowledge must be accompanied by both an increase pull (demand) for innovations and an increase in capacity of the infrastructure to deliver the innovations [11]. To date, most efforts in dissemination research have focused mainly on disseminating innovations, with little emphasis on increasing demand among potential users [27]. Consumers’ preexisting dispositions, preferences, perceptions, capacities, and behaviors determine their response to the innovation and shape their decision-making processes [9]. Partnering with the community is a critical component to translate research into a wider population practice [28-30], and community involvement may enhance the translation and dissemination process [31-34]. In this new model, we use a CBPR approach to bridge the gap between research and practice, as well as academia and community. A successful market depends on the availability of information about the available products and the ability of individuals to access that information, and it can be achieved by using multiple formal and informal media, including newspapers and websites [26]. However, this one-way communication, even if it is repeated through multiple channels, is typically insufficient to move an individual toward a positive action. Persuasion through a two-way communication of social influence has proven more effective [35]. In this project, we used community health advisors (CHAs) who were familiar with the culture, language, and local community, to spread information and generate demand from community members. A social norm marketing campaign in the form of small media, which was found to be effective in promoting CRC screening [36], was conducted to build public awareness and generate demand. This project was approved by the University of Chicago Institutional Review Board (IRB: 052689-0). All procedures followed were in accordance with the ethical standards of the responsible committee on human experimentation, and with the Helsinki Declaration of 1975.

### Creating a Research-Community Dissemination Infrastructure

We invited six Asian-American community-based organizations (CBOs) to join our EBP dissemination community task force during year one. These six CBOs provide direct services to five Asian subgroups (Cambodian, Chinese, Korean, Laotian, and Vietnamese). In Year two, we invited two additional CBOs that serve Filipinos and Southern Asians. Recognizing that the complexity in translating lessons learned from science into practice required a multilevel approach, we also assembled an academic EBP steering committee to accelerate the integration. The academic EBP steering committee consisted of seven different internal departments from the academic institution. Our EBP dissemination community task force and academic EBP steering committee met periodically, separately and jointly, to communicate project progress and disseminate study findings.

### Assessing Community Partners’ Capacity and Readiness for Evidence-Based Practice Dissemination

The adoption of EBP depends on the capacity of CBOs to implement and sustain particular activities. An initial assessment of each CBO’s capacity, based on the strength of its leadership, existing programs and outreach, strategic plan, financial stability, and support staff, was important for the implementation of dissemination-related activities. Assessments were completed via a paper-and-pencil survey. Not all items contributed equally to the implementation process, so the research team used a Delphi method to develop a score system to consider the significance of each item. The Delphi expert panel included three academic experts and two community experts. The research team also developed an EBP readiness survey based on literature review and expert inputs. The EBP readiness survey assessed the stage of our CBO partners along a continuum of readiness. The 7-item EBP readiness survey measured four domains related to the readiness to adopt EBP. These four domains were (1) a defined need for EBP, (2) readiness for change in organizational culture, (3) time, resources, and personnel for EBP, and (4) ability to sustain the change. Under each item, CBO partners chose the stage statement that described their current state of readiness for EBP. Findings from the organizational capacity assessment and EBP readiness survey were used to inform the dissemination process and capacity building.

### Conducting Consumer Research

Although evidence-based CRC screening guidelines have been widely communicated through mainstream media channels, these messages likely have limited reach among AAs because dissemination methods and content are not culturally sensitive or language-specific. Consumer research is one of the significant components of the Market-Orientated Push and Pull Model. Consumer research allows researchers to listen to their consumers to ensure that marketing strategies are well designed, well implemented, and effective by centering the product on their consumers, keeping the product relevant, and understanding which of the product’s *constellation of benefits* to prioritize. In this project, we used a two-step approach to conduct consumer research in our partner communities.

First, we conducted 8 focus groups with a total of 72 participants from 7 Asian subgroups (Cambodian, Chinese, Filipino, Korean, Laotian, South Asian, and Vietnamese) to elicit beliefs and attitudes related to CRC screening. These focus groups were facilitated by bilingual, bicultural CBO staff members, who participated in a 4-hour training session to enhance their facilitation skills. A focus group guide was developed to ensure consistency across groups. Eligibility criteria for the focus group required that participants were (1) between age 40 and 65, (2) lived in different households, and (3) were capable of giving consent. The focus groups were conducted in participants’ native language and tape recorded. The tape recordings were then translated into English by the facilitator. A team of five people worked together to perform content analysis, using template analysis method based on the Theory of Planned Behavior Model.

Secondly, findings from the focus groups were used to develop an Individual Client Survey. The goal of the Individual Client Survey was to determine behavioral beliefs, normative beliefs, and perceived control beliefs regarding CRC screening within the target population. The development of the Individual Client Survey included (1) developing a pool of survey statements (n=91) from the focus group findings, (2) soliciting feedback from CBO partners (n=11) regarding relevance and cultural appropriateness, and (3) weighting the statements for their relevance and significance. The final survey instrument contained 20 statements and was approved by all community partners. Besides the English version, the final survey instrument was translated into six different languages. The survey used a cross-sectional design with a purposeful (based on age group and sex) and convenient sample. A total of 470 surveys were collected from 7 different Asian communities (Table 1). Findings from the Individual Client Survey were then used to design a dissemination plan, train community health advisors, and develop a social marketing campaign.

**Table 1:** Individual Client Survey participants by Asian subgroups.

Asian Subgroup	n
Cambodian	50
Chinese	55
Filipinos	67
Korean^a^	121
Laotian	70
South Asians	49
Vietnamese	58
Total	470

^a^Two Korean community based organizations participated in the Individual Client Survey.

### Generating Public Awareness Through Social Marketing and Social Influence.

In this project, we used consumer-centered social marketing. Consumer-centered social marketing goes beyond pushing the product to the consumer by building demand for the product. The marketing campaign aimed to address sociocultural norms as well as linguistic barriers regarding CRC screening. Although social marketing campaigns can create public awareness, which is the first step toward taking action, information alone is not enough to prompt interest, shape attitudes, and bring about behavior change. Ultimately, the goal of dissemination is not to simply get the word out, but to take the user from awareness to understanding, to commitment, and then to action. We used CHAs as an influencer, each of whom was a bilingual, bicultural community health professional who understood the social norms of the community. To build on these strengths, and to equip them to carry out this work, all CHAs attended a 6-hour training on CRC screening, motivational interviewing techniques, and the application of Stage of Change Theory.

### Investigating the Effectiveness of Community Health Advisor Intervention in Conjunction with Social Marketing

We will use a multiple baseline design to evaluate the effectiveness of our CHA intervention. If every group shows a similar change after crossing to the intervention condition and does not change at other times, the findings will provide compelling evidence that the changes resulted from the intervention [37]. In addition, multiple baseline design guards the internal validity of the study by ruling out the possibility that a single external event (eg, a celebrity cancer diagnosis) could explain the results. In this project, each community will experience a transition from the baseline condition to the intervention condition, but these transitions will be observed over different time periods. The study procedure is outlined as follows:

A baseline CRC screening education session will be conducted in each community prior to the rollout of the CHA intervention and social marketing campaign.

Partner CBOs will be asked to post event flyers as usual, but to refrain from active recruitment.

Participants at each educational session will be asked to fill out a survey, which includes demographic items and beliefs statements selected from the Individual Client Survey.

Partner CBOs will be divided into two cohorts: cohort 1 (Chinese, Filipino, and Laotian) and cohort 2 (Cambodian, Korean, South Asian, and Vietnamese).

Depending on the cohort assignment, partner CBOs will be asked to implement the CHA intervention and social marketing campaign, or do nothing.

The CHA intervention will be a 12-week long intervention and will be carried out concurrently with the social marketing campaign.

At the end of each intervention period, an educational session will be conducted in each community, and no-cost Fecal Immunochemical Test (FIT) kits will be offered to participants.

Participants who took home a FIT kit will have two weeks to return the kit for testing.

The primary outcomes will be the number of participants in each education session, the number of no-cost FIT kits distributed after the education session, and the number of FIT kits returned. Other secondary outcomes will include beliefs and attitudes in CRC screening and intention to screen within the next 12 months. Beliefs and attitudes regarding CRC screening will be measured using items selected from the Individual Client Survey. Participants will also be asked whether they spoke with the CHA prior to the session. The unit of analysis will be cohort (n=2). Statistical tests will be carried out to assess post-intervention differences within and between cohorts, as well as over time.

### Evaluating the Implementation Process and Translatability Using the RE-AIM Framework.

Glasgow and associates (1999) designed an evaluation framework to assess the impact of interventions based on five factors: *Reach, Efficacy, Adoption, Implementation,* and *Maintenance* (RE-AIM [38]). This framework expands the assessment of interventions beyond efficacy to multiple criteria that are better able to identify the translatability and public health impact of interventions. In this project, the evaluation will focus on organization level measurements and include both qualitative and quantitative data. Quantitative data will be collected through weekly CHA reports. Each CHA will be required to maintain a daily activity log and to submit weekly reports including number of individuals educated, number of group education sessions (>5 participants) conducted, number of printed materials distributed, and number of follow-up encounters. Qualitative data will also be collected during site visits at week 3, week 7, and week 10. This data will include, but are not limited to, compliance to the intervention protocol, barriers, facilitators, and concerns. Post-intervention focus groups with implementation CHAs will be conducted to obtain feedback regarding the implementation process and experience. In-depth interviews with partner CBO leadership will also be conducted to assess the CBPR process, satisfaction with the project, and sustainability of the dissemination infrastructure.

## Results

### Assessing Community Partners’ Capacity and Readiness for EBP Dissemination

Of the seven partner CBOs, only one CBO had an organization capacity weighted score above the 70^th^ percentile, and two CBOs had a weighted score below the 50^th^ percentile. Of the four stages of readiness for EBP dissemination (stage 1 = there is no agenda or promotion for EBP in the organization; stage 4 = totally ready for EBP dissemination), two CBOs were at stage 3, two were between stage 2 and 3, and three were at stage 2. In general, there was some understanding of the needs for EBP, but there was a lack of mechanism in the organization to move EBP forward. Although three of the CBOs included EBPs in their organizational agenda and were specially discussed, they had not yet been promoted by leadership.

### Consumer Research

A convenience sample of 72 AAs participated in 8 focus groups. Most of the participants were female (47/72, 65%), and average age was 55 years. All participants were born outside of the United States, and 20 of 72 (28%) had been in the United States 10 years or less. Although differences were noted across ethnic groups, many respondents were unaware of CRC risk, screening benefits, or screening access. Many respondents attributed CRC to pollution in their home countries, stress of immigrant life, or diet. Respondents from countries with more advanced healthcare systems, such as Korea, were more knowledgeable of screening options.

A total of 470 participants completed the Individual Client Survey from 7 Asian subgroups, with 464 surveys entered for final statistical analysis. Most participants (457/464, 98.5%) were foreign-born, and 222 of 464 (47.8%) had lived in the United States more than 20 years. The average age was 56 years, and 146 of 464 respondents had completed less than 9 years of education. Most participants agreed that early detection of cancer was important (434/464, 93.5%), cancer could happen to anyone (403/464, 86.9%), CRC could be prevented (344/464, 74.1%), and everyone should screen for CRC (389/464, 83.8%). However, 35.8% (166/464) of participants also felt that people were better off not knowing it they had cancer, and 45.5% (211/464) would screen only when they had symptoms. Most participants (402/464, 86.6%) said that they would screen upon their doctor’s recommendation, but approximately half of the participants (231/464, 49.8%) reported that they only saw a doctor when they were sick.

Currently, we are conducting our social marketing campaign and implementing the CHA intervention. Data collection is underway and will conclude March 2016. We expect that analysis and results will be available by June 2016.

## Discussion

There is a limited research base to inform the design of successful action plans for dissemination and implementation EBP, especially in underserved and marginalized populations. Little attention has been paid to contextual factors, as well as what the users really need and want during the dissemination process. Users’ preexisting dispositions, preferences, perceptions, and capacities impact individual decision-making processes. Although several conceptual frameworks have been developed, and are useful for generating hypotheses for future research [11,39,40], we urgently need practical frameworks for developing and testing dissemination approaches. A multi-level approach is needed to accelerate integration of lessons learned from science into community health care. The combination effect of a multilevel intervention may have a synergistic effect greater than the sum of the individual parts of the intervention. Such synergy can occur when a set of necessary conditions must be jointly present for change to take place, or when an intervention at one level facilitates or reinforces an intervention at another [41]. This project targeted three different levels (community, interpersonal, and individual) to accelerate the adoption of CRC screening guidelines. Our community-centered Dissemination and Implementation Model provides a systematic approach that is feasible to implement in real-life settings, even within resource-restricted communities. The model also outlines a complementary role for researchers and community organizations in disseminating EBP. Our focus on partnerships and understanding potential adopters has the potential to produce wanted and sustainable innovations. Finally, our model incorporates social interactions and influences to move individuals from simple awareness, to decisions, and then to positive action. By triggering a demand for evidence-based innovations, we can increase the success of our dissemination efforts and the adoption of EBP among the underserved and vulnerable populations most in need of effective, evidence-based practice.

## Abbreviations

AA: Asian American
CBO: community-based organization
CBPR: community-based participatory research
CHA: community health advisor
CRC: colorectal cancer
EBP: evidence-based practice
FIT: Fecal Immunochemical Test
